# Recovery from alopecia areata in a patient with autoimmune polyglandular syndrome type 3

**DOI:** 10.1530/EDM-14-0084

**Published:** 2015-02-01

**Authors:** Shinya Makino, Takeshi Uchihashi, Yasuo Kataoka, Masayoshi Fujiwara

**Affiliations:** Department of Internal Medicine, Osaka Gyomeikan Hospital, 5-4-8 Nishikujo, Konohana-ku, Osaka, 554-0012, Japan

## Abstract

Recovery from alopecia is rare in autoimmune polyglandular syndrome (APS). A 41-year-old male was admitted to our hospital with hyperglycemia. He developed alopecia areata (AA) 5 months before admission and developed thirst, polyuria, and anorexia in 2 weeks. His plasma glucose level upon admission was 912 mg/dl (50.63 mmol/l) and HbA1c was 13.7%. Although urinary and plasma C-peptide levels showed that insulin secretion was not depleted, anti-insulinoma-associated antigen 2 antibody was present. In addition, measurement of thyroid autoantibodies revealed the presence of Hashimoto's thyroiditis. These findings suggested a diagnosis of APS type 3. The patient has showed signs of improvement with the continuation of insulin therapy. During the successful control of diabetes, he had total hair regrowth within 2–3 months. Human leukocyte antigen typing showed that DRB1*1501-DQB1*0602 and DQB1*0301 were present. Similar cases should be accumulated to clarify the association of APS type 3 with recovery from AA.

**Learning points:**

- Alopecia in diabetic patients is a suspicious manifestation of autoimmune type 1 diabetes.
- Patients with autoimmune type 1 diabetes specifically manifesting alopecia should be further examined for diagnosis of APS.
- Insulin-mediated metabolic improvement may be a factor, but not the sole factor, determining a favorable outcome of alopecia in patients with autoimmune type 1 diabetes.

## Background

Alopecia areata (AA) is a tissue-specific, T cell-mediated autoimmune disease of the hair follicles (1) and is often associated with autoimmune polyglandular syndrome (APS) or autoimmune thyroid disease (AITD). The clinical features of AA are heterogenous with respect to age of onset, extent of involvement, and disease duration. The majority of patients present with limited patches of alopecia on the scalp that regrow spontaneously within 1 year, whereas an estimated 7–10% of patients may experience more extensive and chronic forms of the disease (2). Although the precise prognosis of AA in APS is uncertain, associated autoimmune disease is a possible factor that might predict poor outcomes of AA in APS (1).

Herein, we present a case of APS type 3 with a severe form of AA (76–99% hair loss) that might not be predicted to show an improvement from hair loss (3). However, following the successful control of diabetes, the patient had total hair regrowth within 2–3 months. The patient has shown improvement for both diabetes and AA over the last 5 years.

## Case presentation

**Figure 1:**
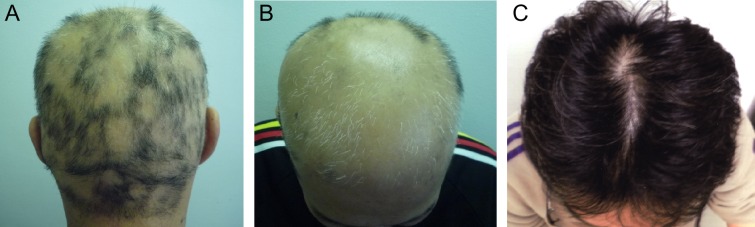
Alopecia areata (AA) in a patient with APS type 3. Approximately 80% of the scalp hair were lost at the time of admission (A and B). He had total hair regrowth within 2–3 months, and his AA has been under control for 5 years. AA at 1.5 years after the initiation of insulin therapy (C).

A 41-year-old male was admitted to our hospital with hyperglycemia on 3rd February 2009. During a health check in 2007, his fasting plasma glucose was 115 mg/dl (6.38 mmol/l) and HbA1c was 6.3%, indicating impaired glucose tolerance (IGT). In September 2008, he abruptly developed AA and complained of upper abdominal pain. He visited another hospital where he was diagnosed with a duodenal ulcer. At this time, his random plasma glucose level was 163 mg/dl (9.05 mmol/l). On 10th December 2008, his random plasma glucose level was elevated to 303 mg/dl (16.70 mmol/l), but he did not receive any anti-diabetic medication. In January 2009, his alopecia gradually worsened and he rapidly developed thirst, polyuria, and anorexia at 2 weeks before admission. He was referred to our hospital on 3rd February 2009. His plasma glucose level upon admission was 912 mg/dl (50.63 mmol/l) and HbA1c was 14.1%. Physical examination on admission showed that his skin and tongue were dry due to dehydration. He showed ∼80% of scalp hair loss (Fig. 1A and B), but his other body hair was normal.

## Investigation

His height was 175 cm and weight was 84.0 kg. His blood pressure was 126/80 mmHg. Laboratory findings revealed a red blood cell count of 509×10^4^/mm^3^, hemoglobin of 16.1 g/dl, and hematocrit of 43.7%. The white blood cell count was 13 500/mm^3^ with 79% neutrophils, and his platelet count was 20.7×10^4^/mm^3^. Serum electrolytes were as follows: Na 129 mEq/l (mmol/l); K 4.7 mEq/l (mmol/l); Cl 92 mEq/l (mmol/l); and Ca 8.4 mg/dl (2.10 mmol/l). Serum blood urea nitrogen was 12.6 mg/dl (4.75 mmol/l); creatinine, 0.73 mg/dl (64.5 μmol/l); and uric acid, 6.8 mg/dl (404.5 μmol/l). Total serum protein was 6.2 g/dl with 63.6% albumin; AST, 85 IU/l; ALT, 135 IU/l; LDH, 251 IU/l; γ-GTP, 97 IU/l; and total cholesterol, 204 mg/dl (5.28 mmol/l). Arterial blood gas analysis had a pH of 7.368; PaO_2_, 81.4 mmHg; PaCO_2_, 40.9 mmHg; HCO_3_, 23.0 mEq/l (mmol/l); and base excess of −2.1 mEq/l (mmol/l).

As shown in Table 1, urinary and plasma C-peptide levels showed that insulin secretion was not depleted. Furthermore, islet cell antibodies, insulin autoantibodies, or anti-glutamic acid decarboxylase antibodies were not detected. However, anti-insulinoma-associated antigen 2 (IA-2) antibodies were present, suggesting autoimmune type 1 diabetes. In addition, an examination of thyroid autoantibodies revealed the presence of Hashimoto's thyroiditis. Plasma cortisol and adrenocorticotropin (ACTH) were normal, indicating no presence of Addison's disease. These findings suggested a diagnosis of APS type 3. HLA typing showed that DRB1*1501-DQB1*0602 and DQB1*0301 were present (Table 2).

**Table 1:** Autoimmune antibodies and basal hormonal levels in a patient with APS type 3 and AA

	**Conventional unit** (normal range)	**SI unit**
Anti-GAD-Ab	<0.4 U/ml (<1.5)	
Anti-IA-2-Ab	1.1 U/ml (<0.4)	
ICA	(–)	
Insulin-Ab	5.1% (<10)	
Anti-nuclear Ab	<×40 (<40)	
Anti-TPO-Ab	26.0 U/ml (<0.3)	
Anti-thyroglobulin-Ab	197.0 U/ml (<0.3)	
Mitochondrial (M2)-Ab	<5 (<7)	
Anti-LKM-1-Ab	<5 (<17)	
C-peptide (plasma)	1.26 ng/ml (0.74–3.48)	0.427 nmol/l
C-peptide (urine)	53.4 μg/day (10.5–167.9)	17.7 nmol/day
TSH	2.24 μIU/ml (0.38–3.64)	2.24 IU/l
Free T_3_	3.2 pg/ml (2.1–4.1)	4.92 pmol/l
Free T_4_	1.1 ng/dl (0.9–1.7)	14.2 pmol/l
ACTH	29 pg/ml (7–56)	6.39 pmol/l
Cortisol	10.2 μg/dl (4.5–21.1)	281.4 nmol/l
Testosterone	4.27 ng/ml (2.01–7.50)	14.8 nmol/l
LH	3.02 mIU/ml (0.79–5.72)	3.02 IU/l
FSH	3.26 mIU/ml (2.00–8.30)	3.26 IU/l
Intact PTH	87.2 pg/ml (10.0–65.0)	9.59 pmol/l

APS, autoimmune polyglandular syndrome; AA, alopecia areata; GAD, glutamic acid decarboxylase; IA-2, insulinoma-associated antigen 2; ICA, islet cell cytoplasmic antibodies; TPO, thyroperoxidase; LKM, liver kidney microsomal.

**Table 2:** HLA DNA typing in a patient with APS type 3 and AA

		
A	110101	260301
B	150101	5603
C	0102	030301
DRB1	1202	1501
DQA1	0102	0601
DQB1	030101	060201

HLA, human leukocyte antigen; APS, autoimmune polyglandular syndrome; AA, alopecia areata. Underline indicates HLA phenotypes.

## Treatment, outcome, and follow-up

Intensive insulin therapy was initiated and plasma glucose levels were normalized in a couple of days. Since the initiation of treatment, the patient has improved following the continuation of insulin therapy. The HbA1c levels are controlled at 6.1–6.5% over 6–8 units/day insulin. He has not developed major diabetic complications such as neuropathy, nephropathy, or retinopathy over the course of disease. Anti-IA-2 antibodies are continuously positive but plasma C-peptide levels are maintained within the normal range, suggesting slowly progressive type 1 diabetes. During successful diabetic control, he had total hair regrowth within 2–3 months. Over the last 5 years, the patient has been in a good condition for both diabetes and AA (Fig. 1C).

## Discussion

APS is characterized by a combination of at least two endocrine organ failures mediated by autoimmune mechanisms. It is classified into four subtypes, namely APS types 1, 2, 3, and 4 (4) (5). Type 3 APS comprises AITD and type 1 diabetes without Addison's disease. Type 3 APS also includes non-endocrine organ-specific autoimmune disorders such as pernicious anemia and/or alopecia. The patient in this case study had type 1 diabetes, Hashimoto's thyroiditis, and AA, but lacked Addison's disease. Thus, the patient could be diagnosed as having type 3 APS.

Type 1 diabetes is classified as fulminant, acute-onset, or slowly progressive, depending on the way of onset and progression (6). This patient already had IGT for at least 1 year before he entered the apparent hyperglycemic state in December 2008. Although he developed hyperglycemic symptoms and was admitted with pre-diabetic ketosis <3 months after the onset of the hyperglycemic state, the presence of IGT for at least 1 year indicated that he should be diagnosed as slowly progressive, but not acute-onset, type 1 diabetes. As plasma C-peptide levels were maintained within the normal range for 5 years, this also supports the diagnosis of slowly progressive type 1 diabetes. In accordance with this, Horie *et al*. (7) reported that the frequency of HLA DRB1*0803-DQB1*0601, a protective haplotype for abrupt-onset type 1 diabetes in Japanese population, was significantly lower in type 1 diabetes without AITD, but not in type 1 diabetes with AITD.

In this patient, total hair regrowth was observed within 2–3 months after the initiation of insulin therapy. Aw & Cheah (8) showed that AA totalis, which developed simultaneously with diabetes mellitus, was improved after insulin therapy, indicating that the recovery from metabolic disturbance was involved in regrowth of the hair. Taniyama *et al*. (9) also reported a case of simultaneous development of type 1 diabetes and AA universalis. However, they found that alopecia did not regress after the metabolic state improved following insulin therapy. Taken together, insulin-mediated metabolic improvement may be one of the number of factors determining the favorable outcome of AA.

There are a number of reports showing that alopecia is a manifestation of APS types 2 and 3 (10) (11) (12) (13). Of these, two studies reported no recovery from alopecia (12) (13), and the others did not describe the outcome of alopecia. Thus, a precise prognosis of AA in APS is uncertain; however, the severity of AA at disease onset and associated autoimmune diseases are possible factors that might predict a poor outcome in AA (1) (3). Tosti *et al*. (3) found that the severity of AA at disease onset was the most important negative prognostic factor in adults, and that patients with >50% of scalp hair loss tended to remain stable or worsen over time. In this regard, this is a very rare case that recovered from a severe form of AA (∼76–99% hair loss) with multiple autoimmune diseases, namely APS type3.

His HLA typing showed that DRB1*1501-DQB1*0602, a protective haplotype for Japanese APS type 3 (14), and DQB1*0301, a susceptible allele for AA (1), were present. Interestingly, another case of APS type 3 with alopecia also had the HLA DRB1*1501-DQB1*0602 haplotype, similar to this patient (10). At present, it is uncertain whether APS type 3 with alopecia in these two patients is associated with this protective haplotype because most reports focusing on alopecia in APS types 2 and 3 lack genetic analyses (11) (12) (13). Therefore, it is important to accumulate similar cases to the one presented herein and such cases should include genetic analysis including HLA typing, to clarify the factors or mechanisms involved in pathogenesis and recovery from AA in APS.

## Patient consent

Written informed consent was obtained from the patient for publication of this case report.

## Author contribution statement

S Makino was the physician responsible for the patient and he reviewed and edited the manuscript. T Uchihashi, Y Kataoka, and M Fujiwara were the patient's physicians.
